# Flake-Shaped Nickel Hydroxide Supported on Carbon Cloth as an Electrochemical Sensor for Efficient Detection of Phosphate Under Neutral Conditions

**DOI:** 10.3390/s25030597

**Published:** 2025-01-21

**Authors:** Haoyu Jiang, Yuhan Hao, Xu Zhao, Feiyang Chen, Chun Zhao, Hui Suo

**Affiliations:** 1 State Key Laboratory of Integrated Optoelectronics, College of Electronic Science and Engineering, Jilin University, Changchun 130012, China; jhy23@mails.jlu.edu.cn (H.J.); haoyh24@mails.jlu.edu.cn (Y.H.); chenfy2222@mails.jlu.edu.cn (F.C.); zchun@jlu.edu.cn (C.Z.); 2 College of Chemistry, Jilin University, Changchun 130012, China; zhaoxu@jlu.edu.cn

**Keywords:** electrochemical sensor, detection of phosphate, neutral condition, Ni(OH)_2_/CC

## Abstract

Although the detection of phosphate can be achieved by nickel hydroxide instead of noble metals, the sensitive detection of phosphate using nickel hydroxide transformed into hydroxy nickel oxide needs to be done under alkaline conditions, which is not environmentally friendly. To solve this problem, we prepared flake-shaped nickel hydroxide-sensitive electrodes supported on carbon cloth using a low-consumption one-step method from the new strategy that the specific adsorption of nickel hydroxide to phosphate can change the electrode’s surface charge distribution and investigated their performance in detecting phosphate under neutral conditions. The specific adsorption of phosphate changed the charge distribution on the surface of the electrode, improved the electron transfer efficiency, and facilitated the valorization of nickel, while the flake-shaped nickel hydroxide provided more active sites for the electrode, which resulted in a good performance of the nickel hydroxide electrode: a sensitivity of 1530 mA mM^−1^ cm^−2^, a detection range of 1–200 μM, and the LOD of 0.59 μM (S/N = 3). This work provides an innovative idea for the determination of phosphate under neutral conditions, which has important practical applications.

## 1. Introduction

Phosphorus is an important element in the atmospheric cycle, the earth’s ecosystem, and the living system, widely distributed in the natural environment, plant, and animal tissues [1]. Phosphorus content is an important indicator for environmental pollution monitoring and food quality control [2], closely related to environmental pollution control and people’s health as well as safety [3,4].

Phosphorus exists in water bodies mainly in two forms, organic and inorganic: organic phosphorus (OP) includes monophosphate, phospholipids, and phosphoproteins. Inorganic phosphorus (IP) includes orthophosphates and condensed phosphates [5]. Phosphate, the most important inorganic form of phosphorus [6], plays an important role for cell division and complex energy transformations in zooplankton. However, it will affect the growth and reproduction of zooplankton when the phosphate content is too low [7]. If the phosphate content in the aquatic ecosystem is too high, more than 0.5 mg/L [4], which will cause eutrophication of the water body and disrupt the balance of the water body ecosystem. Controlling phosphate levels in drinking water is essential to maintain good water quality and protect people’s health and safety. The World Health Organization (WHO) states that the international standard for drinking water is 1 mg/L [6]. Exceeding or far exceeding this standard will be hazardous to human health and safety. If people consume too much phosphate, it will lead to hyperphosphatemia (adult serum PO_4_^3−^ level > 44.6 mg/L) and also cause kidney disease, leading to serious health problems [8,9].

Therefore, it is important for the prediction of the eutrophication in water bodies and the protection of human health and safety to detect the content of phosphate. Currently, some laboratory methods for phosphate detection are chromatography [10], colorimetric [11], fluorescence detection [12], spectrophotometry [13], electrochemical methods, and chemiluminescence. Among which, colorimetric, spectrophotometric, and electrochemical methods can be used for on-site testing. In comparison to traditional laboratory methods, they can determine the phosphate content sensitively and accurately, with a fast response time and good repeatability. However, colorimetric and spectrophotometric methods involve toxic reagents and complicated pretreatment, while electrochemical methods are simple and easy to operate, with increasing popularity among researchers and scholars.

Among the electrochemical methods used for detection are precious metals [14], carbon materials [15,16], biological enzymes [17], and metal (hydro)oxides [18,19]. Precious metals have strong catalytic properties but are expensive. Carbon materials have a large surface area, but their fatigue resistance is poor. Biological enzymes have good selectivity but poor stability. Metal hydroxides have good catalytic properties and low cost but poor stability. However, among them, metal hydroxide has been used by more and more scholars to detect phosphate because of its good catalytic activity and low cost.

Nickel hydroxide, a p-type semiconductor, has excellent electrochemical activity and high catalytic properties. Ni also has various valence states that allow it to be varied. Because of these properties, it is used in a variety of applications: electrical energy storage, catalysts, capacitors, and sensing. The use of nickel hydroxide-based electrodes for the detection of phosphate has been reported in several papers during the century. In 2010, Chen et al. (Zhongxing University, Taichung City, Taiwan) found that barrel nickel-plated electrodes (Ni-BPE) can be activated to form Ni(OH)_2_/NiO(OH) film in alkaline medium (0.1 M NaOH solution), which can trigger the adsorption of phosphate on the surface of the electrode, which would inhibit the glucose electrocatalytic oxidation current. Based on this, Chen et al. developed a method for phosphate detection [20]. In 2019, Sotoudeh et al. (Purdue University) first prepared nickel oxide/hydroxide (NiO/NiOOH)-PrC electrodes on low-cost nickel oxide-modified screen-printed carbon (PrC) electrodes using a simple anodic electrodeposition method and found NiOOH reacts with H_2_PO_4_^−^ to produce nickel phosphate, triggering a change in the current response, which was used to detect phosphate [18]. In 2020, He et al. from Nanjing University of Technology (NUIST) reported a one-step hydrothermal method for the detection of phosphate by nanosheets and nanoflowers of nickel hydroxide (NHH/NF) sensitive electrodes prepared by a one-step hydrothermal method. It is to detect phosphate by using the addition of HPO_4_^2−^ to promote the formation of NiO_2_ from NiOOH, which causes the change of current [21]. In 2022, Xu et al., Tohoku University, showed that specifically modified nickel electrodes, at pH 4, showed current response changes with the addition of H_2_PO_4_^−^ [3].

Although research scholars have reported a large amount of literature on the detection of phosphate based on nickel hydroxide electrodes. However, researchers have mostly started from the strategy that nickel hydroxide is easily transformed into nickel hydroxide by itself in an alkaline environment, and nickel hydroxide can be specific for phosphate detection to prepare nickel hydroxide-based electrodes for phosphate detection under alkaline conditions. Or the high activity of some specific electrodes in acidic environments is utilized for phosphate detection. Thus, these have led researchers and scholars to work on choosing either strong alkaline or strong acidic environments for phosphate detection. However, under neutral conditions, nickel hydroxide is not easily converted to nickel hydroxide, and specific electrodes do not easily remain active, which has made it technically difficult to detect phosphate using the above strategies. Thus, no researcher has detected phosphate based on nickel hydroxide electrode in a neutral environment.

Moreover, alkaline and acidic working environments are not friendly to the environment in field testing, so it is necessary and important to find a method that can detect phosphate under neutral conditions. Layered double hydroxides (LDHs), with compositional and structural tunability, interlayer anion exchangeability, tunable particle size and distribution of intercalated assemblies, and high catalytic properties, are widely used in a variety of applications. In recent years, it has been reported in the literature that LDHs have greater affinity for anions with higher charge densities [22], relatively high specific surface area, relatively strong anion exchange capacity, relatively weak interlayer binding capacity [23], and exchange of interlayer pristine anions with the outside world by a simple ion exchange method [24]. Researchers and scholars have conducted numerous studies on these properties of LDHs and have come to the following conclusion: The anion competition adsorption strength of LDHs is ranked as follows: phosphate > carbonate ≈ sulfate > fluoride > chloride > bromide > nitrate. Therefore, based on the conclusions of the researchers, we speculate that nickel hydroxide-based (double) hydroxides, with the addition of phosphate, will also cause the exchange of ions on their surfaces, which will lead to a change in the current, thus indirectly detecting phosphate.

In this study, in order to achieve the sensitive detection of phosphate ions under neutral conditions via electrochemical methods. We prepare hydroxide-sensitive electrodes, which can be used for the sensitive detection of phosphate ions by taking advantage of their strong anion exchange capacity and the fact that the exchange of phosphate will change the distribution of charges on the surface of hydroxide electrodes, which will lead to a change in the current. In the present study, a nickel hydroxide/carbon cloth-sensitive electrode was prepared by a low-consumption, one-step water bath method based on a carbon cloth electrode substrate. The modification of the carbon cloth by Ni(OH)_2_ provides the electrode with the ability to specifically adsorb phosphate, and the flake-shaped Ni(OH)_2_ provides the electrode with more active sites and improves the electron transfer rate. The synergistic effect of these advantages above gives Ni(OH)_2_/CC excellent performance in phosphate detection, including a low detection limit, high sensitivity, and a wide detection range.

## 2. Materials and Methods

### 2.1. Chemical Reagent

All chemical reagents used in this experiment were analytically pure and did not require further purification. Sodium sulfate (Na_2_SO_4_), potassium chloride (KCl), sodium chloride (NaCl), and potassium ferricyanide (K_3_[Fe(CN)_6_]) were purchased from Beijing Chemical Factory Co., Ltd. (Beijing, China). Ammonium chloride (NH_4_Cl), trisodium phosphate (Na_3_PO_4_), zinc sulfate (ZnSO_4_), ammonia, magnesium sulfate heptahydrate (MgSO_4_·7H_2_O), and potassium hydroxide (KOH) were purchased and obtained from Sinopharm Chemical Reagent Co., Ltd. (Shanghai, China). Glucose (C_6_H_12_O_6_), nickel nitrate hexahydrate (Ni(NO_3_)_2_·6H_2_O), and urea (CO(NH_2_)_2_) were purchased from Yungtay Chemical Reagents Ltd. (Tianjin, China).

### 2.2. Pretreatment of Carbon Cloth

Pretreatment of the carbon cloth was aimed at removing impurities such as grease and organic matter from its surface and improving its hydrophilicity. The steps of processing were as follows: firstly, the carbon cloth was flatly cut into small pieces of 1.5 × 2 cm^2^, after which it was ultrasonically cleaned using toluene, acetone, ethanol, and deionized water sequentially for a total of three times, for five minutes each time. Then, the washed carbon cloths were immersed in a diluted 1 mM hydrochloric acid solution for three hours. Finally, the carbon cloths were rinsed to neutrality several times using deionized water and stored in deionized water for later use.

### 2.3. Preparation of Ni(OH)_2_/CC Sensitive Electrodes

Using a simple and low-consumption one-step water bath method, Ni(OH)_2_/CC sensitive electrodes were prepared. Firstly, 1.4539 g of Ni(NO_3_)_2_·6H_2_O was dissolved in 50 mL of deionized water, stirring for 20 min, and then 12 mL of the above solution was taken at a time and added into several 50 mL centrifuge tubes, respectively, and after that, 60 μL of concentrated ammonia was dropped into the centrifuge tubes by using a pipette gun, and then stirred uniformly, obtaining the precursor solution for water bath preparation. After that, the treated carbon cloth was put into the centrifuge tubes, and the preparation was carried out in a water bath at 45 °C for 5 h. After the preparation, the obtained sensitive electrodes were rinsed with ethanol and deionized water several times in sequence. Finally, the Ni(OH)_2_/CC sensitive electrode was dried for 2 h at 60 °C.

### 2.4. Characterization and Electrochemical Testing

The study of the surface morphology and structure of the prepared electrodes was done by field emission scanning electron microscopy (SEM, JEOL-JEM-6700F, JEOL, Tokyo, Japan). The study of the crystal structure of the prepared electrodes was done by X-ray diffraction spectroscopy (XRD, D8ADVANCE, Bruker, Karlsruhe, Germany Cu Kα source (λ = 1.54 Å)). The surface chemistry and valence of the prepared electrodes were done using X-ray photoelectron spectroscopy (XPS, ESCALAB-250, Thermo Fisher, Waltham, MA, USA). Electrochemical characterizations and testing were done on an electrochemical workstation.

The electrochemical characterization and testing in this paper were done on an electrochemical workstation (CHI 760D, Shanghai Chenhua Instrument Co., Shanghai, China). A typical three-electrode system, Ni(OH)_2_/CC as the working electrode (0.5 × 0.5 cm^2^ area of the working electrode immersed in 0.5 M NaCl electrolyte), Pt sheet (1.5 × 1.5 cm^2^) as the counter electrode, and SCE (saturated calomel electrode) as the reference electrode. Electrochemical testing was carried out in 0.5 M NaCl electrolyte at room temperature, and the cyclic voltammetry test uses a scan rate of 50 mV/s.

## 3. Results and Discussion

### 3.1. Characterization of Ni(OH)_2_/CC Sensitive Electrodes

**Figure 1:**
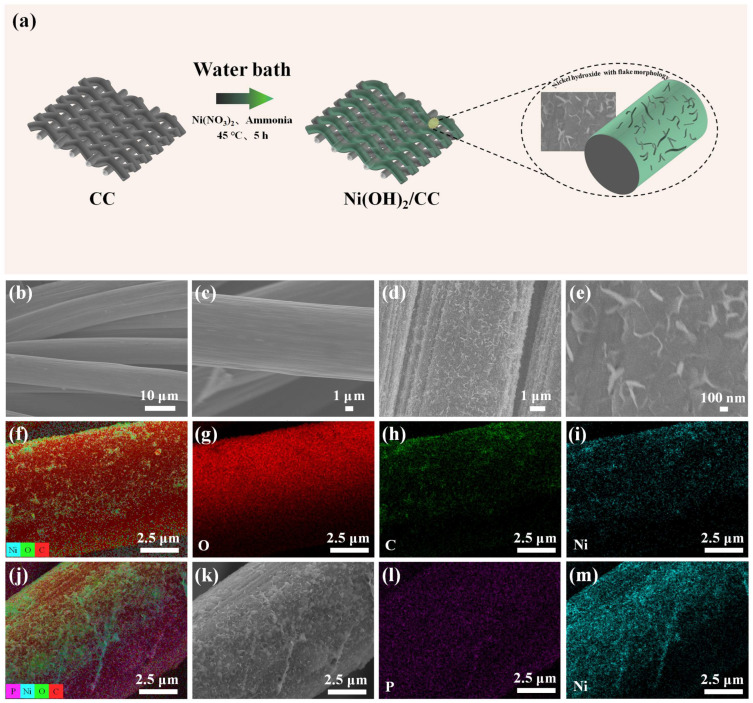
(**a**) Schematic illustration of the Ni(OH)_2_/CC fabrication process. (**b**,**c**) SEM image of bare CC; (**d**,**e**) SEM images of the Ni(OH)_2_/CC; EDS elemental mapping of (**f**) Ni(OH)_2_/CC (before detecting phosphate), (**g**) O, (**h**) C, (**i**) Ni, (**j**) Ni(OH)_2_/CC (after detecting phosphate), (**l**) P, (**m**) Ni, (**k**) SEM image of Ni(OH)_2_/CC (after detecting phosphate).

As shown in Figure 1a, a flow chart of the preparation of Ni(OH)_2_/CC electrodes is illustrated. The electrode with Ni(OH)_2_ modified carbon cloth was obtained by water bath preparation on the surface of interleaved, smooth, and impurity-free bare carbon cloth. Among them, the precursor solution was obtained from Ni(NO_3_)_2_ and ammonia in a certain proportion (see Experimental Section 2.3 for details), with a water bath time of 5 h and a temperature of 45 °C. It can be seen that the nickel hydroxide on the surface of the carbon cloth presents thin flakes, which are uniformly and non-accumulatively distributed on the surface of the carbon cloth.

The morphology and microstructure of the bare CC and Ni(OH)_2_/CC electrode surfaces were characterized by scanning electron microscopy (SEM). Figure 1b,c shows the SEM photographs of bare CC, which show that the carbon cloth consists of many interlaced carbon fibers with smooth and unadulterated surfaces, and the average diameter is 10 μm. We observed that the morphology of the electrode, obtained by the water bath method, modified with Ni(OH)_2_ is shown in Figure 1d,e. In the high-magnification images, Ni(OH)_2_ appears in the form of flakes, uniformly and without aggregation on the surface of the carbon cloth. It can be observed that the flakes here are of a very thin state, unlike the larger interlaced flakes found in other literature [25]. The thin state is not conducive to agglomeration and enhances the conductivity of the electrode and improves the electron transfer rate as well as its adhesion ability. These are favorable to its electrochemical properties. In addition, we analyzed the elemental distribution of the Ni(OH)_2_/CC electrode by EDS elemental spectra. As shown in Figure 1f–i and Figure S1, the distributions of the elements C, O, and Ni are approximately the same, and all of them cover the surface of the carbon cloth fibers uniformly, which, together with the SEM results, proves that the preparation of Ni(OH)_2_ on CC is successful.

Because we use the process that phosphate is adsorbed by Ni(OH)_2_ and exchanged with the original anions on the surface of Ni(OH)_2_ which leads to a change in the charge distribution on the surface of the electrode and then causes a change in the current response to indirectly detect phosphate. Therefore, a certain amount of phosphate should exist on the surface of the prepared Ni(OH)_2_/CC electrode after the detection of phosphate. Therefore, we performed EDS elemental spectroscopy analysis on the after-test electrode, as shown in Figure 1j–m. Compared with Figure 1d, it can be seen that on the surface of the after-test electrode, a layer of white lumps was attached to the original flake-shaped Ni(OH)_2_ surface, which was uniformly distributed without large accumulations. In combination with Figure 1l, it can be seen that this should be the phosphate adsorbed on the electrode surface, which changes the surface morphology of the electrode and also further affects the charge distribution on the electrode surface, affecting the electron transfer rate.

**Figure 2:**
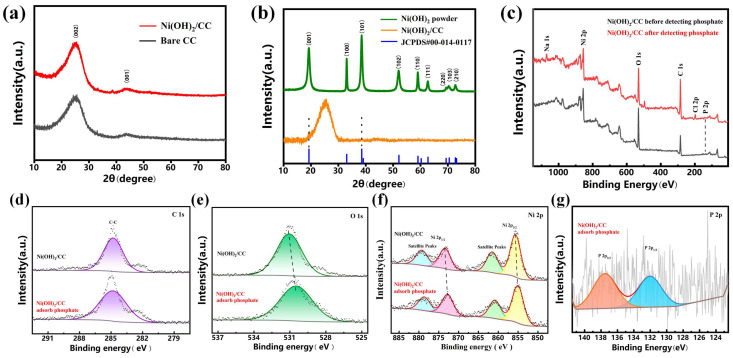
(**a**) XRD pattern of Ni(OH)_2_/CC and bare CC electrodes. (**b**) XRD patterns of powders and Ni(OH)_2_/CC obtained with longer preparation time. XPS spectra of Ni(OH)_2_/CC before and after electrode testing: (**c**) survey spectrum, (**d**) C 1s, (**e**) O 1s, (**f**) Ni 2p, and (**g**) P 2p.

According to Figure 2a, the crystalline phases of bare carbon cloth and Ni(OH)_2_/CC can be studied and analyzed by X-ray diffraction spectroscopy (XRD). For the XRD spectrum of bare CC, two broad peaks can be seen at 25.04° and 44°, which are due to the reflection of (002) and (001) of hexagonal graphitic carbon (JCPDS No. 75-1621) with typical sp^2^ bonds. Observing the XRD spectrum of Ni(OH)_2_/CC prepared under the optimal preparation conditions, it can be seen that there are little differences with that of the bare CC, and we analyzed it that it is because the Ni(OH)_2_/CC electrode, obtained by the water-bath preparation method, has a thinner Ni(OH)_2_ growing on the surface, and the original characteristic peaks of the carbon cloth (the background peaks) override the characteristic peaks of the Ni(OH)_2_ thus the characteristic peaks of Ni(OH)_2_ could not be measured by XRD. We further characterized the new electrodes obtained at longer preparation times and the powders obtained by water bath preparation to demonstrate the successful preparation of the electrodes, as shown in Figure 2b. Compared with Figure 2a, it can be seen that new faint peaks appear at 19.16° and 38.56°, corresponding to the diffraction of the single-crystal ramps (001) and (101) of Ni(OH)_2_ (JCPDS#00-014-0117), respectively. In addition, these two peaks, which are the same as the corresponding peak positions in the maps obtained from the characterization of Ni(OH)_2_ powder, demonstrate that Ni(OH)_2_ was successfully prepared on the surface of CC. The diffraction peaks of Ni(OH)_2_ powder were clear and intense, indicating that the nano-Ni(OH)_2_ has good crystallinity. In addition, no diffraction peaks of other nickel-containing substances were found, indicating that the prepared product was a single phase and of high purity.

The chemical composition and valence states of the Ni(OH)_2_/CC electrode were analyzed using X-ray photoelectron spectroscopy (XPS). The full spectrum is shown in Figure 2c, which reveals the characteristic peaks of the elements C, O, and Ni. As shown in Figure 2f, in the Ni 2p spectrum, two peaks centered on 855.7 eV and 873.5 eV belong to Ni 2p_3/2_ and Ni 2p_1/2_, respectively [26]. The binding energy gap between Ni 2p_3/2_ and Ni 2p_1/2_ is about 17.8 eV, which is consistent with the results published by previous research scholars [21]. These results demonstrate that elemental Ni exists in the prepared electrodes as a +2 hydroxide state. The peaks centered on 861.4 eV and 873.5 eV belong to the satellite peaks of Ni [27]. In addition, for the O 1s spectrum (Figure 2e), only one peak was fitted, and the fitted peak at 531.05 eV showed the presence of Ni-OH bonds, further confirming the formation of Ni(OH)_2_ [26]. In the carbon 1s spectrum (Figure 2d), only one C-C bond peak belonging to the carbon cloth was found at 284.8 eV. The XPS results correspond to the XRD results, further proving the successful preparation of the self-supported Ni(OH)_2_/CC electrode.

We have also analyzed the XPS characterization of the tested Ni(OH)_2_/CC electrode. The full spectrum is shown in Figure 2c, in which not only characteristic peaks of C, O, and Ni elements, but also the peak positions are consistent with those before the test, can be seen, but also new characteristic peaks of Na, Cl, and P elements appear. The presence of the elements Na and Cl is due to the fact that the electrodes during electrochemical testing work in an environment with an electrolyte of 0.5 M NaCl. There are large amounts of Na^+^ and Cl^−^ in the electrolyte. The principle of the test in this paper is that phosphate will be attached to the electrode surface due to the specific adsorption of nickel hydroxide, which will change the charge distribution on the electrode surface and then trigger the current change. The electrodes prepared in this paper are reusable (see the reproducibility section later), and after each test, the adsorbed phosphate can be washed off by rinsing with deionized water, and the electrodes can be used for testing again. Here, in order to confirm that the change of the current response is due to the adsorption of phosphate, the electrode should not be rinsed with deionized water at the end of the test. This would result in the shedding of the phosphate, making it impossible to characterize it. Therefore, the XPS test was carried out using an electrode that had not been rinsed with deionized water after the test, and it is obvious that NaCl electrolyte must have been left on the surface of the electrode as well, which is where the Na and Cl elements come from. As shown in Figure 2f, in the Ni 2p spectrum, the two peaks centered on 855.1 eV and 872.8 eV belong to Ni 2p_3/2_ and Ni 2p_1/2_, respectively, which are shifted from the previous peaks, and the positions of the Ni^2+^ and Ni^3+^ peaks in the electrode were shifted to lower binding energies after the test. This suggests that the addition and adsorption of phosphate positively affect the change of Ni between divalent and trivalent, which is consistent with the subsequent cyclic voltammetry test results: the adsorption and attachment of phosphate promote the change of valence of Ni. In the carbon 1s spectrum (Figure 2d), only one C-C bond peak belonging to the carbon cloth was found at 284.85 eV. Moreover, for the O 1s spectrum (Figure 2e), the fitted peak at 530.5 eV belongs to −OH.

### 3.2. Electrochemical Behaviors of Ni(OH)_2_/CC

**Figure 3:**
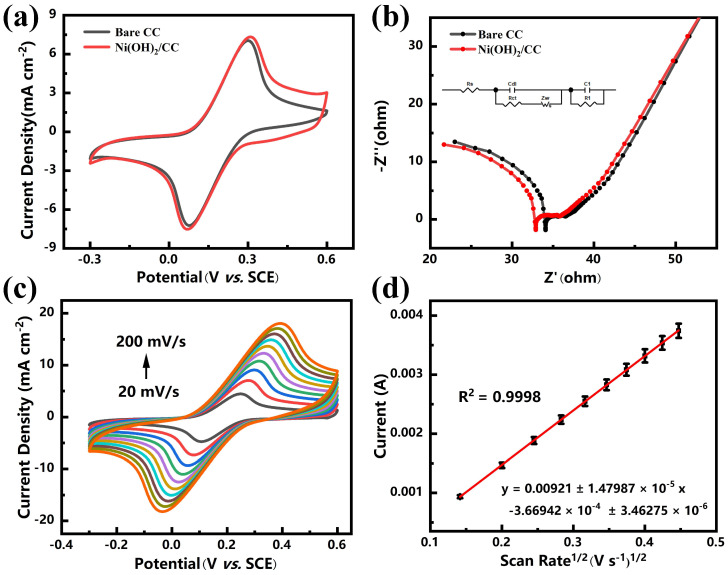
(**a**) CVs and (**b**) EIS spectrum of Ni(OH)_2_/CC and bare CC electrodes in 0.1 M KCl solution containing 5.0 mM [Fe(CN)_6_]^3−/4−^. (**c**) CVs of the Ni(OH)_2_/CC electrode in 0.1 M KCl containing 5.0 mM [Fe(CN)_6_]^3−/4−^ at different scan rates (20–200 mV s^−1^). (**d**) Linear relationship between oxidation peak current and the square root of scan rate.

**Figure 4:**
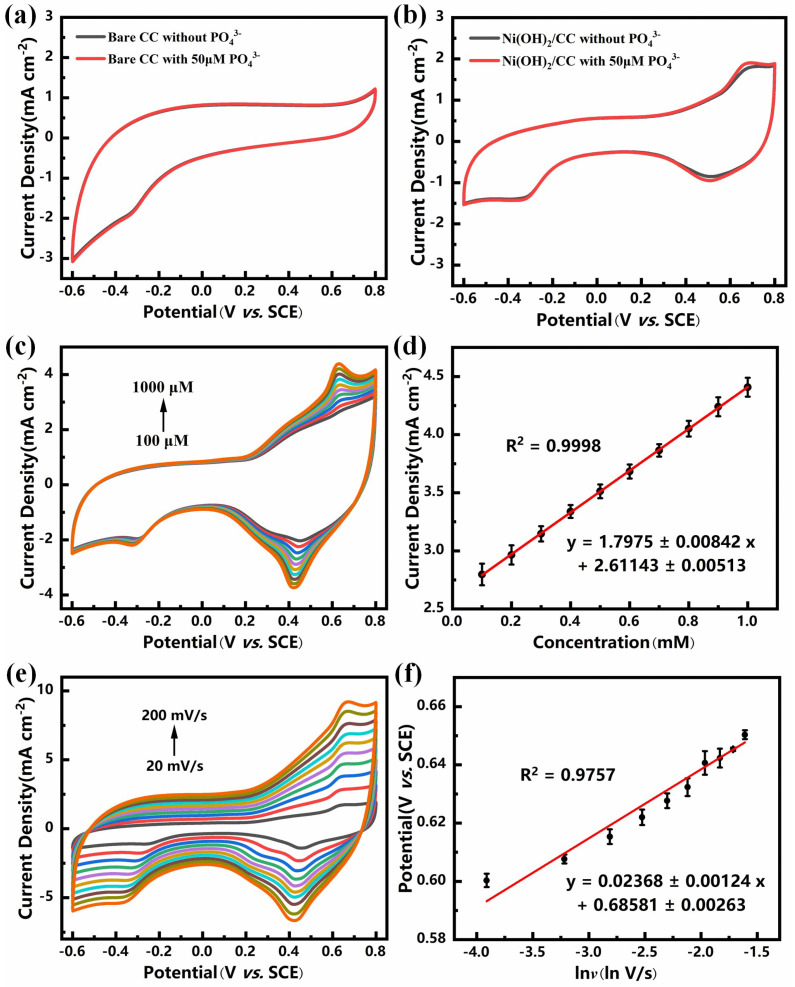
CV curves of the change in current response of bare carbon cloth (**a**) and Ni(OH)_2_/CC (**b**) before and after the addition of phosphate. (**c**) CVs of the Ni(OH)_2_/CC in 0.5 M NaCl under increasing concentration of nitrite solution (0.1–1.0 mmol/L) at a scan rate of 50 mV s^−1^. (**d**) Linear relationship between the oxidation peak current and increasing phosphate concentration. (**e**) CVs of the Ni(OH)_2_/CC in 0.5 M NaCl solution containing 0.5 mM phosphate at different scan rates (20–200 mV s^−1^). (**f**) Linear relationship between oxidation peak potential and the logarithm of scan rate (ln ν).

We investigated the electron transfer kinetic properties as well as the interfacial electrochemical properties of the Ni(OH)_2_/CC electrode by comparing the Ni(OH)_2_/CC electrode with the bare CC electrode. As shown in Figure 3a, it can be seen that the Ni(OH)_2_/CC electrode has a pair of reversible redox peaks similar to the bare carbon cloth electrode. The peak current densities of the two differ little, which is due to the lesser amount of nickel hydroxide obtained by water bath preparation, which does not change the carbon cloth conductivity and specific surface area much, which is reflected in the XRD characterization in the previous. However, the current density of the Ni(OH)_2_/CC electrode at 0.4 V is significantly higher than that of the bare CC, which indicates the modification of the bare CC using Ni(OH)_2_ gives the electrode a larger active specific surface area and higher conductivity. Figure 3b shows the Nyquist plots of Ni(OH)_2_/CC and bare CC electrodes, and the inset shows the corresponding equivalent circuit diagrams. It can be visualized from Figure 3b that the semicircular diameter of the Ni(OH)_2_/CC electrode in the high-frequency part is smaller than that of the bare CC, except that the difference is very small, which is due to the preparation method and the small amount of prepared in situ growth product. Calculation and fitting of the test data yielded R_ct_ values of 22.56 Ω and 23.29 Ω for Ni(OH)_2_/CC and bare CC electrodes, respectively, suggesting that Ni(OH)_2_/CC electrodes have a smaller charge transfer resistance (R_ct_) [28,29]. This suggests that with the modification of Ni(OH)_2_ the charge transfer resistance is reduced and the interfacial electrochemical reaction is facilitated [30]. Because the charge transfer resistance, as reflected by the semicircle diameter of the high-frequency portion of the Nyquist plot, has a smaller value, indicating that the interfacial electrochemical reaction on the electrode surface is promoted. Although the R_ct_ of the nickel hydroxide-modified electrode is only slightly different from that of the bare carbon cloth, which indicates that the interfacial electrochemical reactions of the two surfaces are promoted to the same extent. However, the test results (Figure 4a,b) show that the nickel hydroxide-modified carbon cloth has a change in current response to phosphate that is far from the bare carbon cloth electrode. This suggests that although the nickel hydroxide modification only slightly reduces the value of R_ct_, very good phosphate detection performance is obtained. This side effect shows the irreplaceable role of nickel hydroxide in the testing process.

Cyclic voltammetry tests at different scan rates were performed on the Ni(OH)_2_/CC electrode to investigate the electrochemically active surface area (ECSA) of the electrode, as shown in Figure 3c,d. Figure 3c shows the fitted curve of peak current versus square root of scan rate, and it can be seen that the oxidation peak current (*I_pa_*) of Ni(OH)_2_/CC electrode increases linearly with the square root of scan rate (*v*^1/2^), and the linear regression equation is: *I_pa_ (A)* = 0.00921 ± 1.47987 × 10^−5^ *v*^1/2^ − 3.66942 × 10^−4^ ± 3.46275 × 10^−6^ (R^2^ = 0.9998). Therefore, the ECSA of the electrode can be obtained by calculation based on the Randles-Sevcik equation, which is given as:

*I_pa_* = (2.69 × 10^5^) *n*^3/2^
*A C D*^1/2^
*ν*^1/2^ (1)

In Equation (1), *I_pa_*, *n*, *A*, *C*, *D*, and *v* denote the oxidation peak current (A), the number of transferred electrons (*n* = 1), the electrochemically active surface area of the electrode (cm^2^), the concentration of probe molecules (5 × 10^−6^ mol cm^−3^), the diffusion coefficient (7.60 × 10^−6^ cm^2^ s^−1^), and the scanning rate (V/s), respectively. The ECSA of Ni(OH)_2_/CC and bare CC electrodes was calculated to be 2.08 cm^2^ and 2.01 cm^2^, respectively. This indicates that the doping of Ni(OH)_2_ on the surface of the carbon cloth resulted in increasing the electrochemically active specific surface area of the carbon cloth, adding the electrochemically active sites, and enhancing the charge transport pathways, which would be favorable for the reaction upon the addition of phosphate. In summary, through the study of the electron transfer kinetic properties as well as the interfacial electrochemical properties of the Ni(OH)_2_/CC electrode, the results showed that it has lower charge transfer resistance, higher conductivity, a larger electroactive specific surface area, fast electron transfer kinetics, and excellent electrocatalytic activity, which gives it great potential for the electrochemically sensitive detection of phosphate.

### 3.3. Electrochemical Determination of Phosphate

Before electrochemical testing, we used cyclic voltammetry in different neutral electrolytes to determine the change in current response of Ni(OH)_2_/CC electrodes after adding phosphate. We chose three neutral electrolytes here: sodium sulfate solution, sodium chloride solution, and ABS solution with pH 7. As shown in Figure S3, after adding a certain amount of phosphate to the electrolyte, the Ni(OH)_2_/CC electrode has a significant current response change only in sodium chloride solution with a pair of obvious redox peaks, so here we choose sodium chloride as the electrolyte for future tests. We optimized the parameters of the concentration of sodium chloride solution and the preparation process of Ni(OH)_2_/CC electrode by the water bath method, as shown in Figures S4–S7, so that the optimal concentration of sodium chloride was 0.5 M, and the optimal parameter of Ni(OH)_2_/CC electrode prepared by the water bath was as follows: 60 μL of concentrated ammonia was added to 12 mL of 0.1 M nickel nitrate solution under the condition of 45 °C for 5 h. Subsequent electrodes used for testing were prepared under these optimal preparation conditions.

The effect of Ni(OH)_2_ on the phosphate detection performance was verified by investigating the changes in current response to phosphate at the electrodes, obtained before and after Ni(OH)_2_ modification of carbon cloth by cyclic voltammetry. As shown in Figure 4a,b, it can be seen that the carbon cloth electrode did not exhibit any oxidation or reduction peaks when phosphate was not added, whereas the Ni(OH)_2_/CC electrode showed a pair of obvious redox peaks at 0.65 V as well as at 0.53 V. According to the studies in the published literature, the redox peaks here belong to the variation of Ni of Ni(OH)_2_ itself [31], which is a pair of reversible redox peaks. After the addition of phosphate, no new redox peaks appeared in the carbon cloth electrode, and the current response did not change significantly, while the current response of the redox peaks became higher with the addition of phosphate, which suggests that the modification of the carbon cloth electrode by Ni(OH)_2_ has equipped the modified electrode with the sensitivity to phosphate.

Cyclic voltammetry tests were performed on the Ni(OH)_2_/CC electrode at step-increasing phosphate concentrations as shown in Figure 4c to verify the feasibility of the electrode for the construction of a phosphate sensor. The oxidation peak current density of the Ni(OH)_2_/CC electrode increased linearly with increasing phosphate concentration in the concentration range of 100–1000 μM, and the linear relationship was reflected as *I_pa_* (mA cm^−2^) = 1.7975 ± 0.00842 *C*(mM) + 2.61143 ± 0.00513 with stepwise (shown in Figure 4d), which indicates that the Ni(OH)_2_/CC electrode possesses excellent electrochemical activity and phosphate root sensing ability. This is because the addition of phosphate promotes the variation of Ni in Ni(OH)_2_ which may be due to the fact that the addition of phosphate is adsorbed by Ni(OH)_2_ changing the charge distribution on the surface of the Ni(OH)_2_/CC electrode, and prompting the Ni, which is already valorized, to be more easily valorized, thus resulting in an increasingly strong current response.

In order to investigate the kinetics and mechanism of the reaction of Ni(OH)_2_/CC electrode on phosphate, we investigated the effect of Ni(OH)_2_/CC electrode on the sensitivity of 0.5 mM phosphate detection at different scanning rates, as shown in Figure 4e. It can be seen that the oxidation peak current density (*I_pa_*) increases linearly with the square root of the scan rate (*v*^1/2^), and the linear regression equation is: *I_pa_* (mA cm^−2^) = 0.68548 ± 0.01997 *v*^1/2^ (mV S^−1^) − 1.54193 ± 0.13317 (Figure S8), indicating that the reaction occurring at the electrode belongs to a diffusion-controlled process [32,33].

A linear equation between the oxidation peak potential (*E_pa_*) and the logarithm of the scan rate (*ln v*) was obtained as shown in Figure 4f: *E_pa_* (V) = 0.02368 ± 0.00124 *lnv* (ln V/s) + 0.68581 ± 0.00263. By fitting Laviron’s theoretical model as in Equation (2), the number of transferred electrons involved in the process of current change caused by the addition of phosphate to the Ni(OH)_2_/CC electrode can thus be calculated (n = 1) [34].

(2) $$E_{Pa}=E^{\theta }+(\frac{RT}{\alpha nF})ln(\frac{RTK^{\theta }}{\alpha nF})+(\frac{RT}{\alpha nF})ln\nu$$

In the formula, *E^θ^* is the standard electrode potential (V), *α* is the electron transfer coefficient, *K^θ^* is the standard rate constant (s^−1^) for the electrochemical oxidation of phosphate, and *T*, *R*, and *F* are the absolute temperature (298.15 K), the universal gas constant (8.314 J/(K mol)), and Faraday’s constant (96,485 C/mol), respectively. The value of *αn* was determined from the slope of *E_Pa_* with *ln ν* as 1.087. The number of transferred electrons (n) for the rate-determining step of the electrochemical oxidation of phosphate at the Ni(OH)_2_/CC electrode was calculated to be 1, which is in agreement with the previous report [35]. Based on the above results, the redox mechanism of nitrite on Ni(OH)_2_/CC electrode can be deduced as Equation (3) [36]:

(3) $${\mathrm{Ni}}^{2+}\;⇄{\mathrm{Ni}}^{3+}+e^{-}$$

For deriving the formula, it is simply the change of Ni between divalent and trivalent.

### 3.4. Sensitive Determination of Phosphate

**Figure 5:**
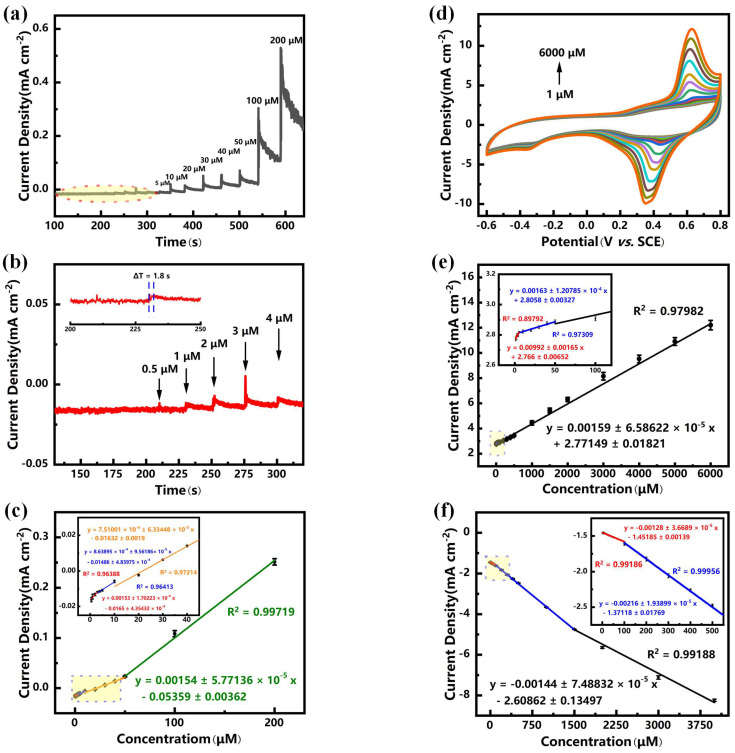
(**a**) Amperometry curve of the Ni(OH)_2_/CC toward phosphate at 0.67 V. (**b**) The expanded plots of the parts labeled in (**a**). (**c**) Linear graph of the current response versus phosphate concentration. (**d**) Cyclic voltammetry curves of Ni(OH)_2_/CC toward phosphate. (**e**) Linear plot of current response at oxidation peak versus phosphate concentration. (**f**) Linear plot of current response at the reduction peak versus phosphate concentration.

We investigated the performance of the sensitive electrodes for the linear detection of phosphate under the timed-current method, and we chose the potential at the oxidation peak in the cyclic voltammetry test as the potential in the timed-current method for the test. As shown in Figure 5a,b. It can be seen that, with the addition of different concentrations of phosphate, the current responses of the Ni(OH)_2_/CC electrode all increased sharply, showing a gradual stepwise upward trend, and reached the maximum value in a very short time (1.8 s), which indicates that the nitrite diffused rapidly to the vicinity of the surface of the Ni(OH)_2_/CC electrode and was adsorbed rapidly, altering the charge distribution of the surface of the electrode, causing the electron transfer, and leading to the current response change. The above phenomena demonstrated the excellent electrocatalytic activity, large electrochemically active surface area, and superior electron transfer capability of the Ni(OH)_2_/CC electrode. As shown in Figure 5c, the current response exhibited a good linear relationship with the nitrite concentration at the low concentration ranging over 1–4 μM: *I_p__a_* (mA cm^−2^) = 0.00153 ± 1.70223 × 10^−4^ *C* (μM) − 0.0165 ± 4.35432 × 10^−4^ (R^2^ = 0.96388), and the high sensitivity was 1530 μA mM^−1^ cm^−2^; at the middle concentration ranging over 4–10 μM, the linear equation was *I_pa_* (mA cm^−2^) = 0.000863895 ± 9.56186 × 10^−5^ *C* (μM) − 0.01486 ± 4.83975 × 10^−4^ (R^2^ = 0.96413), and the high sensitivity was 863.895 μA mM^−1^ cm^−2^; at the high concentration ranging over 10–50 μM, the linear equation was *I_pa_* (mA cm^−2^) = 0.000751001 ± 6.33448 × 10^−5^ *C* (μM) − 0.01632 ± 0.0019 (R^2^ = 0.97214), and the high sensitivity was 751 μA mM^−1^ cm^−2^; at the higher concentration ranging over 50–200 μM, the linear equation was *I_pa_* (mA cm^−2^) = 0.00154 ± 5.77136 × 10^−5^ *C* (μM) − 0.05359 ± 0.00362 (R^2^ = 0.99719), and the high sensitivity was 1540 μA mM^−1^ cm^−2^. From these, the sensitivity of the Ni(OH)_2_/CC electrode is 1530 μA mM^−1^ cm^−2^, and the limit of detection (LOD) of the Ni(OH)_2_/CC electrode for phosphate is as low as 0.59 μM (S/N = 3), which is lower than the nationally defined threshold for eutrophication of the water body (0.5 mg/L [4]). However, we found that using the amperometry method to study the sensitivity of the Ni(OH)_2_/CC electrode for the detection of phosphate, in the low concentration range (1–50 μM), the current response changed rapidly with the addition of phosphate and stabilized within a short period of time, whereas in the middle and high concentration range (100–200 μM), although the current response changes rapidly with the addition of phosphate, it does not stabilize but rather decreases over time, which is detrimental to the accuracy of the assay. We speculate that this is because although phosphate is adsorbed on the electrode surface, there is a limit to the amount of phosphate that can be strongly adsorbed on the electrode surface under stirring conditions, so vigorous stirring dislodges the extra phosphate, leading ultimately to a sustained decrease in the current response.

Therefore, we further used cyclic voltammetry to investigate the sensitivity of the Ni(OH)_2_/CC electrode for the detection of phosphate. We added different concentrations of phosphate solution in 0.5 M NaCl solution within the potential interval of −0.6 V–0.8 V and observed the change of current response of the Ni(OH)_2_/CC electrode. As shown in Figure 5d, the current response of the electrode increases stepwise as the phosphate concentration increases stepwise. In addition, we also noticed that the oxidation peak and reduction peak potentials were shifted to lower potentials with the increase of phosphate concentration in the electrolyte, which indicates that Ni(OH)_2_/CC changes its surface charge distribution after adsorption of phosphate, with an increase in the negative charge, which decreases the potential required for the Ni valence change cycle, making the change of valence more easily realized. Based on the test results, we obtained a linear relationship between the oxidation peak current response and the phosphate concentration over a wide range of 1–6000 μM (Figure 5e): at the low concentration ranging over 1–5 μM: *I_pa_* (mA cm^−2^) = 0.00992 ± 0.00165 *C* (μM) + 2.766 ± 0.00652 (R^2^ = 0.89792), and the high sensitivity was 9920 μA mM^−1^ cm^−2^; at the middle concentration ranging over 5–50 μM, the linear equation was *I_pa_* (mA cm^−2^) = 0.00163 ± 1.20785 × 10^−4^ *C* (μM) + 2.8058 ± 0.00327 (R^2^ = 0.97309), and the high sensitivity was 1630 μA mM^−1^ cm^−2^; at the high concentration ranging over 50–6000 μM, the linear equation was *I_pa_* (mA cm^−2^) = 0.00159 ± 6.58622 × 10^−5^ *C* (μM) + 2.77149 ± 0.01821 (R^2^ = 0.97982), and the high sensitivity was 1590 μA mM^−1^ cm^−2^. The sensitivity of the Ni(OH)_2_/CC electrode was calculated to be 9920 μA mM^−1^ cm^−2^, the result is superior to that obtained under amperometric methods. We also investigated the linear relationship between the current response at the reduction peak and the phosphate concentration (Figure 5f): at the low concentration ranging over 1–100 μM: *I_pa_* (mA cm^−2^) = −0.00128 ± 3.6689 × 10^−5^ *C* (μM) − 1.45185 ± 0.00139 (R^2^ = 0.99186), and the high sensitivity was 1280 μA mM^−1^ cm^−2^; at the middle concentration ranging over 100–1500 μM, the linear equation was *I_pa_* (mA cm^−2^) = −0.00216 ± 1.93899 × 10^−5^ *C* (μM) − 1.37118 ± 0.01769 (R^2^ = 0.99956), and the high sensitivity was 2160 μA mM^−1^ cm^−2^; at the high concentration ranging over 1500–6000 μM, the linear equation was *I_pa_* (mA cm^−2^) = −0.00144 ± 7.48832 × 10^−5^ *C* (μM) − 2.60862 ± 0.13497 (R^2^ = 0.99188), and the high sensitivity was 1440 μA mM^−1^ cm^−2^. The sensitivity of the Ni(OH)_2_/CC electrode was calculated to be 1280 μA mM^−1^ cm^−2^. This suggests that either the oxidation or reduction peaks are capable of being used for highly sensitive detection of phosphate, which makes cyclic voltammetry testing much more efficient. Comparing the performance parameters of the prepared Ni(OH)_2_/CC electrode with those of other phosphate-sensitive electrodes reported previously (shown in Table 1), the Ni(OH)_2_/CC electrode exhibited outstanding properties such as a wide detection range, low LOD, high sensitivity, and fast response time, which predicts a promising future for Ni(OH)_2_/CC sensors in the quantitative detection of phosphate.

In summary, the Ni(OH)_2_/CC electrode prepared by a one-step and low-consumption simple water bath method has good electrochemical detection performance for phosphate, which is attributed to the following aspects. Firstly, carbon cloth, a flexible material with high electrical conductivity, was used as the electrode substrate, ensuring better electrical conductivity and electron transfer ability of the prepared electrode. Secondly, Ni(OH)_2_ was grown directly on the carbon cloth substrate, which not only allowed Ni(OH)_2_ to be tightly connected to the carbon cloth but also avoided the use of conductive additives and polymer binders and also resulted in the strong mechanical stability of the prepared electrodes, lowered the interfacial charge-transfer impedance, facilitated the rapid transfer of electrons, and improved the electrode’s current response. Third, Ni(OH)_2_ has a specific adsorption capacity for phosphate, which allows phosphate to be exchanged with anions on the surface of the Ni(OH)_2_ electrode more rapidly, which in turn alters the charge distribution and promotes changes in the current response. All of the above makes the Ni(OH)_2_/CC electrode possess excellent electrochemical detection performance of phosphate.

### 3.5. Anti-Interference, Stability, and Reproducibility of Ni(OH)_2_/CC

**Figure 6:**
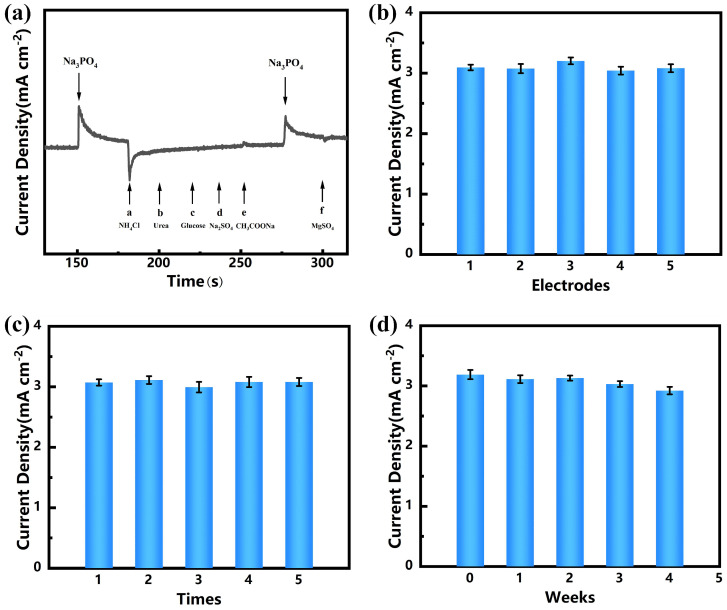
(**a**) Amperometry curve of Ni(OH)_2_/CC towards 20 μM PO_4_^3−^ and 1.0 mM interferents (a–f: NH_4_Cl, urea, glucose, Na_2_SO_4_, CH_3_COONa, MgSO_4_) at 0.67 V. (**b**) Comparative histogram of peak current of 0.1 mM phosphate oxidation by five independent Ni(OH)_2_/CC. (**c**) Comparative histogram of peak current of a Ni(OH)_2_/CC for five consecutive tests on 0.1 mM PO_4_^3−^. (**d**) Comparative column diagram of peak current for 0.1 mM phosphate oxidation on Ni(OH)_2_/CC following 0–4 weeks of storage.

In order to cope with the complexity of practical applications, the immunity of Ni(OH)_2_/CC electrodes has been investigated. As shown in Figure 6a, an inrush current is generated after the injection of 20 μM of phosphate, and the current response changes rapidly and maintains stability quickly. Meanwhile, when the concentration of common interferents is 50 times that of the phosphate concentration (1 mM), the current does not change significantly after the addition of the rest of the interferents, except for NH_4_Cl, which leads to a weakening of the current response. The results indicate that the Ni(OH)_2_/CC electrode prepared in the water bath in this paper has good anti-interference properties and can be used for the quantitative analysis of phosphate in real samples. As shown in Figure 6b, we investigated the reproducibility of the Ni(OH)_2_/CC electrode by performing CV tests on five electrodes prepared under the same conditions with an RSD of 1.41%, which demonstrated that the Ni(OH)_2_/CC electrode has good reproducibility. In addition, we investigated the current response of the Ni(OH)_2_/CC electrode to 0.1 mM phosphate with an RSD of 1.97% for five consecutive times (as shown in Figure 6c), which proved that the Ni(OH)_2_/CC electrode has excellent stability. As shown in Figure 6d, we evaluated the long-term stability of the Ni(OH)_2_/CC electrode by comparing the Ni(OH)_2_/CC electrode response current to 0.1 mM phosphate over 0–4 weeks, and it can be seen that the electrode’s response to the peak current over 0–4 weeks is basically the same, with an RSD of 3.35%. The above tests show that the Ni(OH)_2_/CC electrode has good immunity to interference, repeatability, and long-term stability, which ensure the reliability of the phosphate electrochemical sensor constructed with the Ni(OH)_2_/CC electrode for repeated, long-term use in practical applications.

### 3.6. Actual Sample Testing

The usefulness of Ni(OH)_2_/CC sensitive electrodes for the determination of phosphate in drinking water has been demonstrated by the standard addition method. Drinking water is not required to be pretreated and can be used directly for quantitative measurements. We prepare the electrolyte for testing by combining drinking water and deionized water in a ratio of 1:9 for actual sample testing. Cyclic voltammetry is used in the tests. The voltage range was −0.6 to 0.8 V, and the scan rate was 50 mV/s. Each assay was repeated three times to ensure the reliability of the results. As shown in Table 2, the recoveries ranged from 98.7% to 102.1% with a relative standard deviation (RSD) of less than 6.5%. These results indicate that the Ni(OH)2/CC sensitive electrode is capable of detecting phosphate in real samples.

## 4. Conclusions

In summary, we propose a simple and low-consumption one-step water bath method to grow flake-shaped Ni(OH)_2_ in situ on carbon cloth to obtain a Ni(OH)_2_/CC sensitive electrode as an efficient phosphate detection sensor. The modification of the carbon cloth by Ni(OH)_2_ provided the sensitive electrode with the ability of adsorption of phosphate, providing more active sites and a larger specific surface area. In addition, a thin layer of nickel hydroxide effectively increases the carrier mobility and improves the electron transfer efficiency, thus enhancing its phosphate detection capability. With the synergistic effect of the above advantages, the prepared Ni(OH)_2_/CC electrode possesses excellent phosphate sensing performance, a wide detection range (1–200 μM), high response sensitivity (1530 mA mM^−1^ cm^−2^), a low LOD (0.59 μM (S/N = 3)), as well as good immunity to interference, reproducibility, and stability. In addition, a new phosphate detection method is creatively proposed in this paper, which makes use of the properties possessed by hydroxide to sensitively detect phosphate under neutral conditions, which is environmentally friendly. In addition, the prepared electrode has potential applications in the detection of phosphate in drinking water samples. These highlight the potential of Ni(OH)_2_/CC in the field of phosphate detection and provide a pioneering thought for subsequent research.

## Figures and Tables

**Table 1:** Comparison of Ni(OH)_2_/CC with other phosphate sensors.

Electrode Materials	Sensitivity (μA mM^−1^ cm^−2^)	Linear Range (μM)	LOD (μM)	Working Condition	Reference
NiO/NiOOH-PrC	−78.48 mV dec^−1^ (the response slope)	1–10^5^	1	C_8_H_6_O_4_ (pH = 4.4)	[18]
Ni-BPE	-	1–10^3^	0.3	0.1 M NaOH (pH = 13)	[20]
NHH/NF	210 87	10–14,000 14,000–5 × 10^5^	0.42	NaOH (pH ≈ 11)	[21]
Ni-PME	−81.0 mV dec^−1^ (the response slope)	0.1–10^6^	-	(pH = 4)	[3]
Ni(OH)_2_/CC	1530	1–200	0.59	0.5 M NaCl (pH = 7)	This work

**Table 2:** Electrochemical detection of nitrite in drinking water.

Actual Samples	Initial (μM)	Added (μM)	Found (μM)	Recovery (%)	RSD (%, n = 3)
Drinking water	0	10	10.21	102.1	6.21
		20	19.74	98.7	2.94

## Data Availability

Data are contained within the article and Supplementary Materials.

## Supplementary Materials

The following supporting information can be downloaded at: https://www.mdpi.com/article/10.3390/s25030597/s1. Figure S1: EDS spectrum of the Ni(OH)_2_/CC electrode (before the test); Figure S2: EDS spectrum of the Ni(OH)_2_/CC electrode (after the test); Figure S3: CV graphs of current response change of Ni(OH)_2_/CC electrode before and after phosphate addition in different electrolytes; Figure S4: Different NaCl concentrations (a–e: 0.1 M, 0.3 M, 0.5 M, 0.7M, 1M) and their effect on the electrochemical response of the Ni(OH)_2_/CC electrode to 0.1 M phosphate in the electrolyte; Figure S5: Different temperatures (a–d: 45 °C, 40 °C, 50 °C, 60 °C) and their effect on the electrochemical response of the Ni(OH)_2_/CC electrode to 0.1 M phosphate in the electrolyte; Figure S6: Different times (a–d: 4 h, 5 h, 6 h, 7h) and their effect on the electrochemical response of the Ni(OH)_2_/CC electrode to 0.1 M phosphate in the electrolyte; Figure S7: Different ammonia concentrations (a–d: 30 μL, 60 μL, 75 μL, 90 μL) and their effect on the electrochemical response of the Ni(OH)_2_/CC electrode to 0.1 M phosphate in the electrolyte; Figure S8: Linear relationship between oxidation peak current and the square root of scan rate of the Ni(OH)_2_/CC in 0.5 M NaCl containing 0.5 mM phosphate at different scan rates (20–200 mV s^−1^); Figure S9: (a) CVs of the bare CC 0.1 M KCl containing 5.0 mM [Fe(CN)_6_]^3-^ at different scan rates (20–200 mV s^−1^). (b) Linear relationship between oxidation peak current and the square root of scan rate; Figure S10: (a) CVs of five pieces of independent Ni(OH)_2_/CC electrodes towards 0.1 mM phosphate in 0.5 M NaCl at a scan rate of 50 mV s^−1^. (b) CVs of five consecutive tests of a piece Ni(OH)_2_/CC electrode towards 0.1 mM phosphate in 0.5 M NaCl at a scan rate of 50 mV s^−1^. (c) CVs of Ni(OH)_2_/CC electrode before and after 4 weeks of storage in the presence of 0.1 mM phosphate in 0.5 M NaCl at a scan rate of 50 mV s^−1^.

## References

[1] Wei H. (2022). Electrochemical Detection of Phosphate in Highly Saline and Turbid Waters. Ph.D. Thesis.

[2] Xu J., Gao Z., Dou X., Song Y.Y. (2021). Needle-like Co_3_O_4_ nanoarrays as a dual-responsive amperometric sensor for enzyme-free detection of glucose and phosphate anion. J. Electroanal. Chem..

[3] Xu K., Wu B., Wan J., Li Y., Li M. (2022). A potentiometric phosphate ion sensor based on electrochemically modified nickel electrode. Electrochim. Acta.

[4] Li S.N., You Y., Hu W.G., Gao G.J., Jiang X.Y., Yu J.G. (2023). A sensitive single-layered graphene oxide-based sensor for electrochemical sensing of phosphate anion. Process Saf. Environ. Prot..

[5] Jin J. (2014). Response of Phytoplankton to Phosphorus and Nutrient Dynamics in Typical Sea Area. Ph.D. Thesis.

[6] Sari S.R., Tominaga M. (2023). Progress and current trends in the electrochemical determination of phosphate ions for environmental and biological monitoring applications. Anal. Sci..

[7] Walworth N.G., Fu F.X., Webb E.A., Saito M.A., Moran D., Mcllvin M.R., Lee M.D., Hutchins D.A. (2016). Mechanisms of increased Trichodesmium fitness under iron and phosphorus co-limitation in the present and future ocean. Nat. Commun..

[8] Forano C., Farhat H., Mousty C. (2018). Recent trends in electrochemical detection of phosphate in actual waters. Curr. Opin. Electrochem..

[9] Ano J., Assémian A.S., Yobouet Y.A., Adouby K., Drogui P. (2019). Electrochemical removal of phosphate from synthetic effluent: A comparative study between iron and aluminum by using experimental design methodology. Process Saf. Environ. Prot..

[10] Benito J.Q., Rosario R., Thorsten R. (2006). Determination of Phosphoric Acid Mono- and Diesters in Municipal Wastewater by Solid-Phase Extraction and Ion-Pair Liquid Chromatography-Tandem Mass Spectrometry. Anal. Chem..

[11] Liu W., Du Z., Qian Y., Li F. (2013). A specific colorimetric probe for phosphate detection based on anti-aggregation of gold nanoparticles. Sens. Actuators B Chem..

[12] Ganjali R.M., Hosseini M., Memari Z., Faridbod F., Norouzi P., Goldooz H., Badiei A. (2011). Selective recognition of monohydrogen phosphate by fluorescence enhancement of a new cerium complex. Anal. Chim. Acta.

[13] Abbas D.N.M. (2003). Solid Phase Spectrophotometric Determination of Traces of Arsenate and Phosphate in Water Using Polyurethane Foam Sorbent. Anal. Lett..

[14] Zhao G., Fozia Wen H., Dai Z., Nie Y., Jiang J., Xu X., Ying M., Hu Z., Xu H. (2022). Preparation of a Phosphate Ion-Selective Electrode Using One-Step Process Optimized with Response Surface Method and its Application in Real Sample Detections. Electrocatalysis.

[15] Giordano G.F., Freitas V.M., Schleder G.R., Santhiago M., Gobbi A.L., Lima R.S. (2021). Bifunctional Metal Meshes Acting as a Semipermeable Membrane and Electrode for Sensitive Electrochemical Determination of Volatile Compounds. ACS Appl. Mater. Interfaces.

[16] Mostafa S., Alireza S. (2022). An electronic nose based on carbon nanotube -titanium dioxide hybrid nanostructures for detection and discrimination of volatile organic compounds. Sens. Actuators B Chem..

[17] Samet Kilic M., Korkut S., Hazer B. (2017). Novel Enzymatic Rhodium Modified Poly(styrene-g-oleic amide) Film Electrode for Hydrogen Peroxide Detection. Electroanalysis.

[18] Sedaghat S., Jeong S., Zareei A., Peana S., Glassmaker N., Rahimi R. (2019). Development of a nickel oxide/oxyhydroxide-modified printed carbon electrode as an all solid-state sensor for potentiometric phosphate detection. New J. Chem..

[19] Shooshtari M., Vollebregt S., Vaseghi Y., Rajati M., Pahlavan S. (2023). The sensitivity enhancement of TiO_2_-based VOCs sensor decorated by gold at room temperature. Nanotechnology.

[20] Cheng W.L., Sue J.W., Chen W.C., Chang J.L., Zen J.M. (2010). Activated nickel platform for electrochemical sensing of phosphate. Anal. Chem..

[21] He J., Sun H., Dai J., Wang H., Yu L., Zhou W., Shao Z. (2020). In situ growth of nanoflake and nanoflower-like Ni hydrated hydroxide on the surface of Ni foam as a free-standing electrode for high-performance phosphate detection. J. Hazard. Mater..

[22] Guo Y., Zhu Z., Qiu Y., Zhao J. (2012). Adsorption of arsenate on Cu/Mg/Fe/La layered double hydroxide from aqueous solutions. J. Hazard. Mater..

[23] You Y., Vance F.G., Zhao H. (2001). Selenium adsorption on Mg–Al and Zn–Al layered double hydroxides. Appl. Clay Sci..

[24] Caporale A., Pigna M., Dynes J., Cozzolino V., Zhu J., Violante A. (2011). Effect of inorganic and organic ligands on the sorption/desorption of arsenate on/from Al–Mg and Fe–Mg layered double hydroxides. J. Hazard. Mater..

[25] Zhao X., Zhou G., Qin S., Zhang J., Wang G., Gao J., Suo H., Zhao C. (2024). In Situ Preparation of Metallic Copper Nanosheets/Carbon Paper Sensitive Electrodes for Low-Potential Electrochemical Detection of Nitrite. Sensors.

[26] Zhang L., Xie S., Gu J., Wang X. (2023). Fabrication of core–shell Pt–Ni(OH)_2_ nanosheets on Ni foam and investigation on its detection performance of ammonia–nitrogen in lake and sea water. J. Mater. Sci. Mater. Electron..

[27] Zhang L., Li H., Gu J., Zhao X., Wang X. (2022). Facile one-step synthesis of Pt/Ni(OH)_2_ nanoflakes as sensitive electrode for detection of ammonia–nitrogen in drinking water. Mater. Lett..

[28] Vilian A.T.E., Umapathi R., Hwang S.-K., Huh Y.S., Han Y.-K. (2021). Pd-Cu nanospheres supported on Mo_2_C for the electrochemical sensing of nitrites. J. Hazard. Mater..

[29] Nguyen L.D., Doan T.C.D., Huynh T.M., Dang D.M.T., Dang C.M. (2021). Thermally reduced graphene/nafion modified platinum disk electrode for trace level electrochemical detection of iron. Microchem. J..

[30] Chen L., Zheng J. (2022). Two-step hydrothermal and ultrasound-assisted synthesis of CB/NiCo_2_S_4_@CeO_2_ composites for high-sensitivity electrochemical detection of nitrite. Microchem. J..

[31] Liu Y., Li J., Liu Y., Wang M., Liu K., Cui H. (2024). Revealing the key factors determining Ni(OH)_2_’s electrochemical performance and hereby establishing a carbon shell-protected structure as high-performance electrode material for supercapacitors. J. Energy Storage.

[32] Yang Y., Lei Q., Li J., Hong C., Zhao Z., Xu H., Hu J. (2022). Synthesis and enhanced electrochemical properties of AuNPs@MoS_2_/rGO hybrid structures for highly sensitive nitrite detection. Microchem. J..

[33] Sundaresan P., Lee T.Y. (2022). Facile synthesis of exfoliated graphite-supported cobalt ferrite (Co_1.2_Fe_1.8_O_4_) nanocomposite for the electrochemical detection of diclofenac. Microchem. J..

[34] Li R., Li F., Zhe T., Li M., Liu Y., Wang L. (2021). Three-dimensional (3D) hierarchical structure engineering of AuNPs/Co(OH)(2) nanocomposite on carbon cloth: An advanced and efficient electrode for highly sensitive and specific determination of nitrite. Sens. Actuators B Chem..

[35] Zhe T., Li R., Wang Q., Shi D., Li F., Liu Y., Liang S., Sun X., Cao Y., Wang L. (2020). In situ preparation of FeSe nanorods-functionalized carbon cloth for efficient and stable electrochemical detection of nitrite. Sens. Actuators B Chem..

[36] Huang W., Ge L., Chen Y., Lai X., Peng J., Tu J., Cao Y., Li X. (2017). Ni(OH)_2_/NiO nanosheet with opulent active sites for high-performance glucose biosensor. Sens. Actuators B Chem..

