# Supplementary material for: Flake-Shaped Nickel Hydroxide Supported on Carbon Cloth as an Electrochemical Sensor for Efficient Detection of Phosphate Under Neutral Conditions
Source: Sensors (Basel). 2025 Jan 21;25(3):597. doi: 10.3390/s25030597 (PMC11820762; doi:10.3390/s25030597)
Supplement: Supplementary file 1 [file sensors-25-00597-s001.zip › sensors-3427791-supplementary.pdf]

Supplementary Material

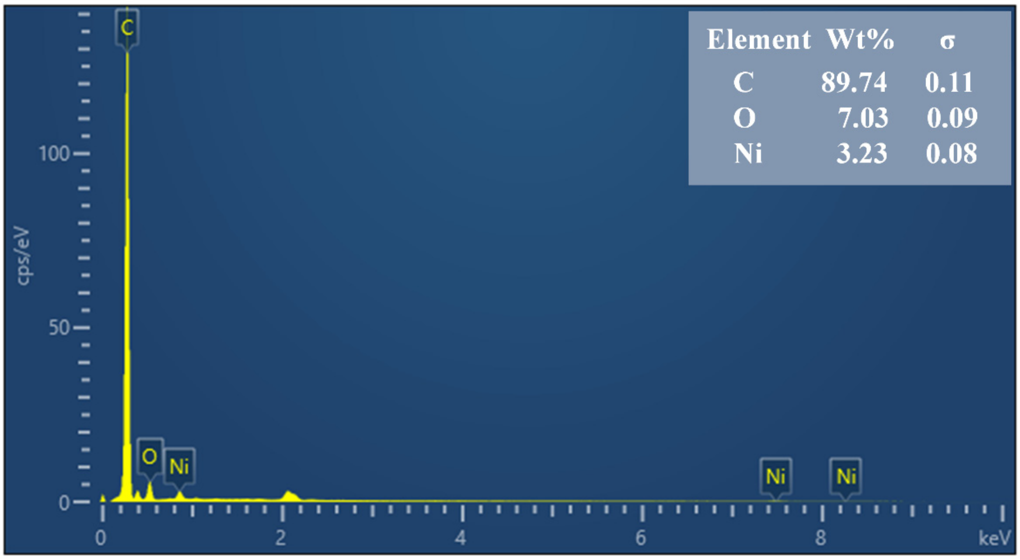

Figure S1. EDS spectrum of the $Ni(OH)_2$/CC electrode (before the test).

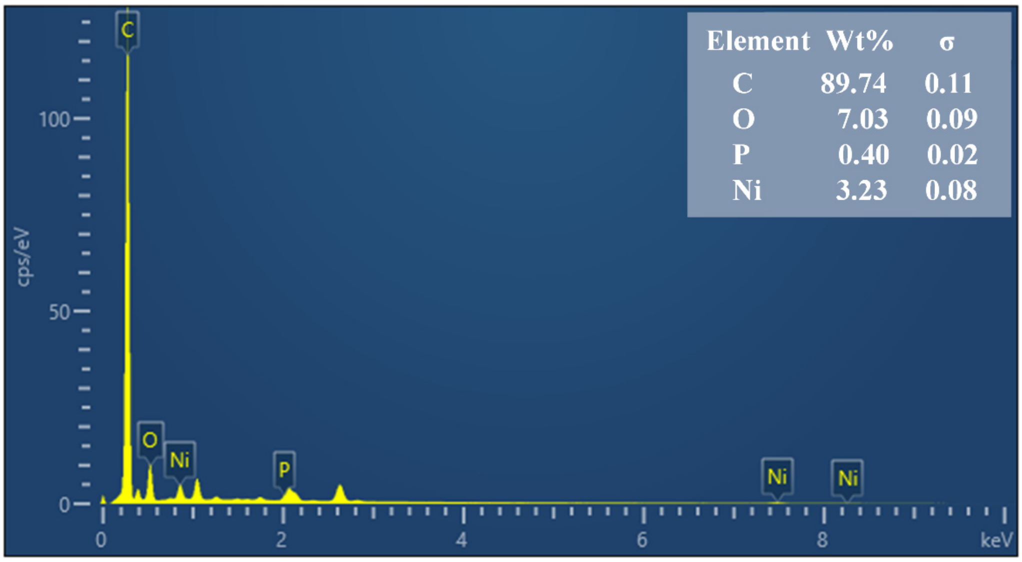

Figure S2. EDS spectrum of the $Ni(OH)_2$/CC electrode (after the test).

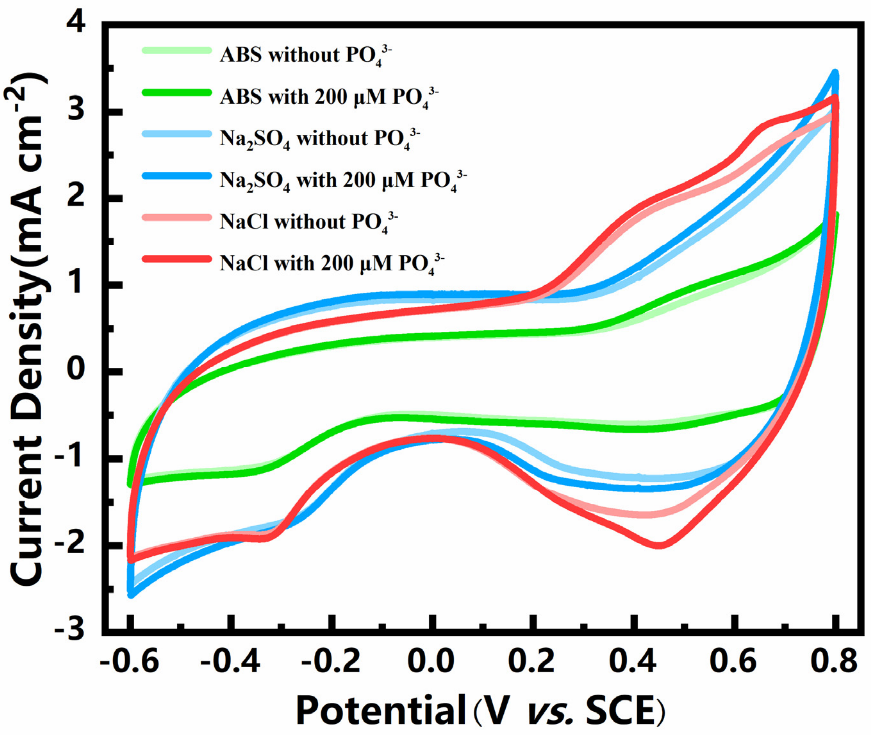

Figure S3. CV graphs of current response change of Ni(OH)2/CC electrode before and after phosphate addition in different electrolytes.

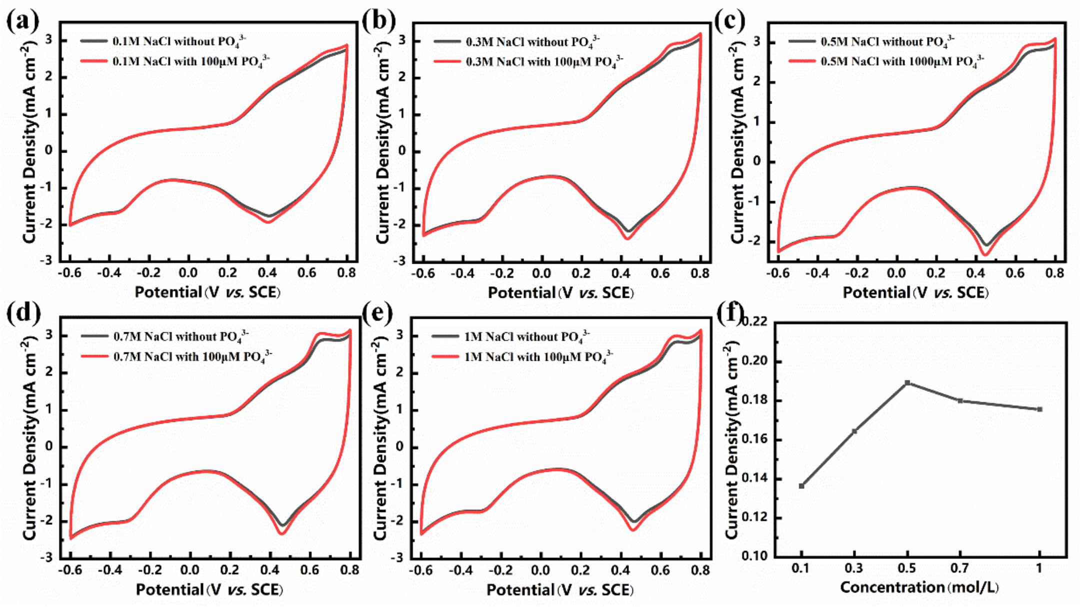

Figure S4. Different NaCl concentrations（a–e: 0.1 M, 0.3 M, 0.5 M, 0.7M, 1M） and their effect on the electrochemical response of the Ni(OH)2/CC electrode to 0.1 M phosphate in the electrolyte.

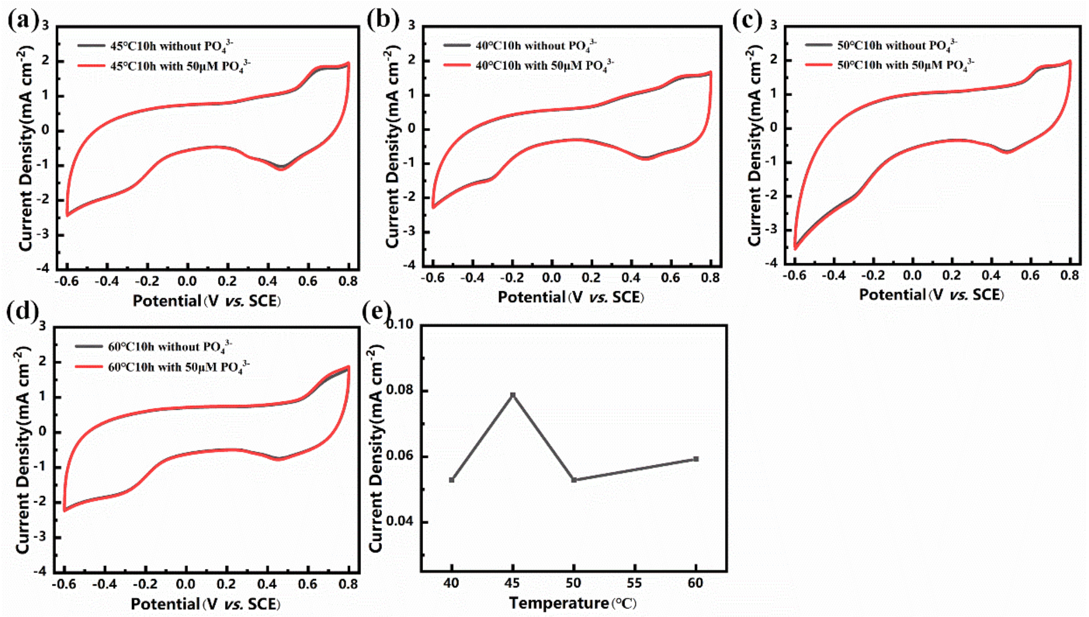

Figure S5. Different temperatures（a–d: 45°C, 40°C, 50 °C, 60°C）and their effect on the electrochemical response of the Ni(OH)$_2$/CC electrode to 0.1 M phosphate in the electrolyte.

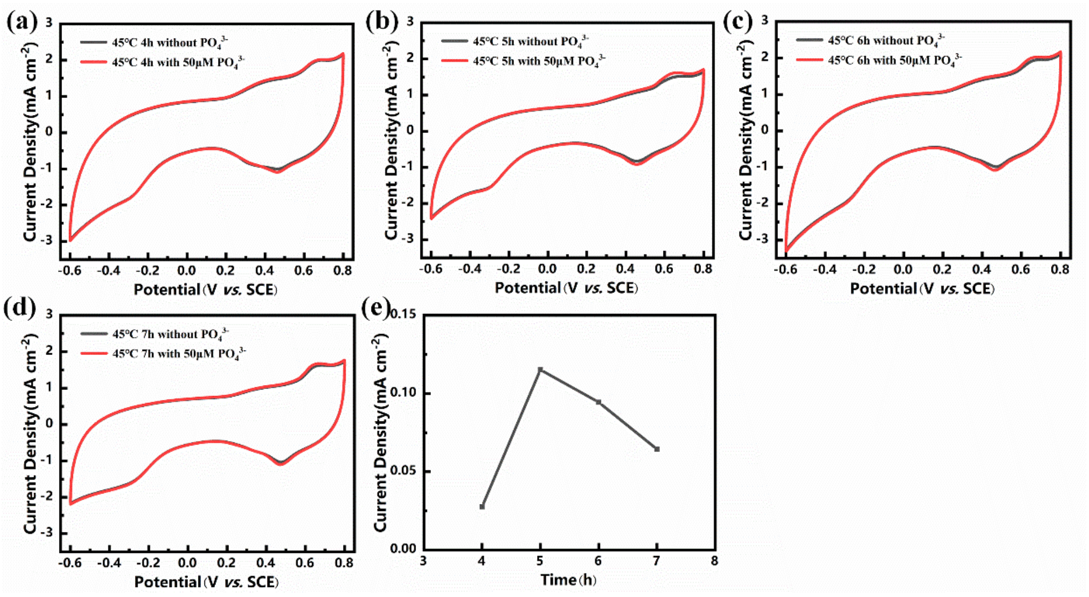

Figure S6. Different times（a–d: 4 h, 5 h, 6 h, 7h）and their effect on the electrochemical response of the Ni(OH)$_2$/CC electrode to 0.1 M phosphate in the electrolyte.

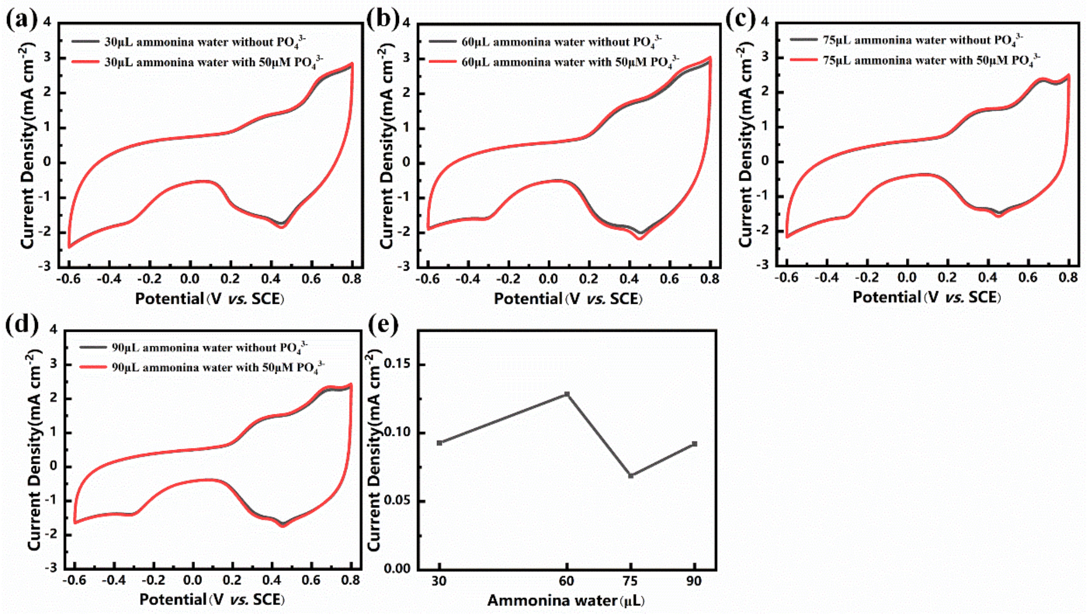

Figure S7. Different ammonia concentrations（a–d: 30 μL, 60 μL, 75 μL, 90 μL）and their effect on the electrochemical response of the Ni(OH)2/CC electrode to 0.1 M phosphate in the electrolyte.

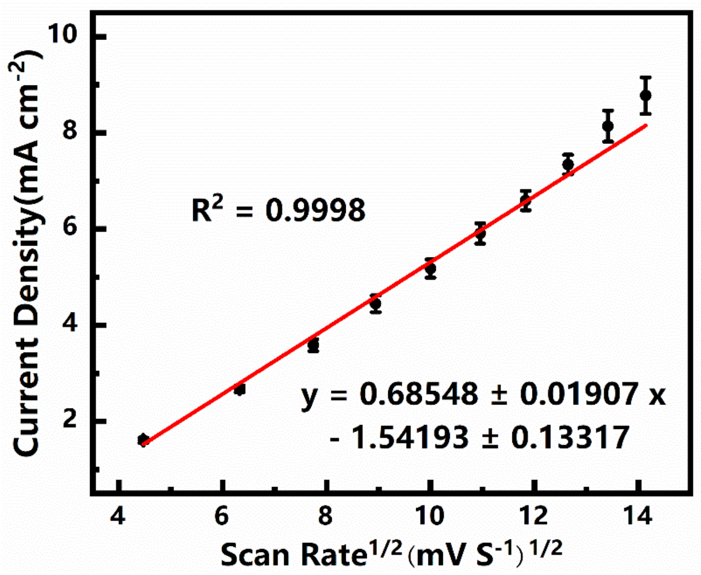

Figure S8. Linear relationship between oxidation peak current and the square root of scan rate of the Ni(OH)2/CC in 0.5 M NaCl containing 0.5 mM phosphate at different scan rates (20–200 mV s-1).

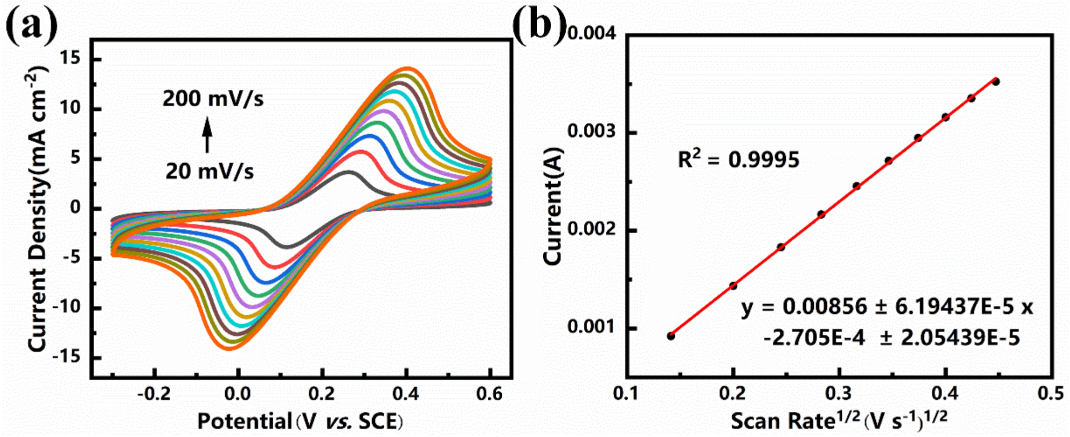

Figure S9. (a) CVs of the bare CC 0.1 M KCl containing 5.0 mM $[Fe(CN)_6]^{3-}$ at different scan rates (20–200 mV s^{-1}). (b) Linear relationship between oxidation peak current and the square root of scan rate.

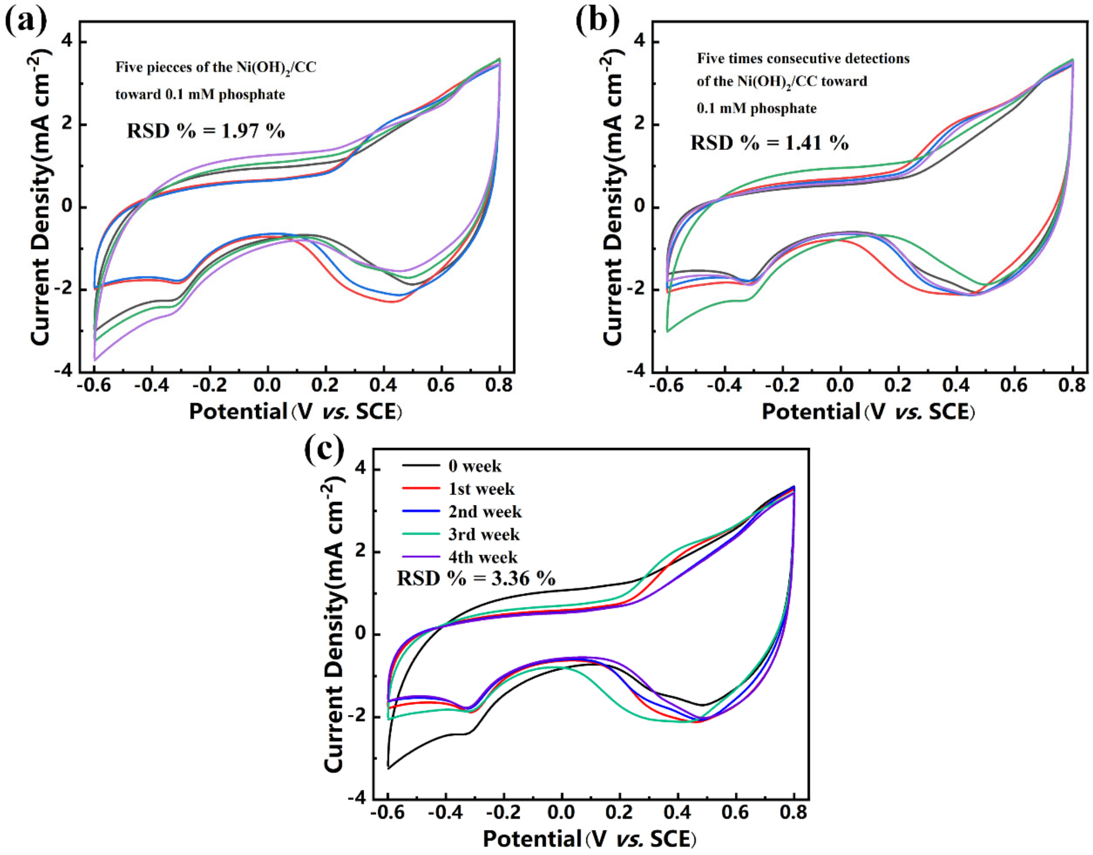

Figure S10. (a) CVs of five pieces of independent $Ni(OH)_2$/CC electrodes towards 0.1 mM phosphate in 0.5 M NaCl at a scan rate of 50 mV s^{-1}. (b) CVs of five consecutive tests of a piece $Ni(OH)_2$/CC electrode towards 0.1 mM phosphate in 0.5 M NaCl at a scan rate of 50 mV s^{-1}. (c) CVs of $Ni(OH)_2$/CC electrode before and after 4 weeks of storage in the presence of 0.1 mM phosphate in 0.5 M NaCl at a scan rate of 50 mV s^{-1}.
